# Pro-adrenomedullin as a clinical predictor after cardiac surgery

**DOI:** 10.1186/cc11970

**Published:** 2013-03-19

**Authors:** J Van Fessem, F De Graaf, J Van Paassen, S Arbous

**Affiliations:** 1 LUMC, Leiden, the Netherlands

## Introduction

Pulmonary complications after cardiac surgery like ARDS are frequent and linked to high mortality [1]. Pro-adrenomedullin (pro-ADM) has a possible role in the development of ARDS [2] and a positive correlation between levels of pro-ADM and inflammation was found [3]. In this study we investigated whether intraoperative and postoperative pro-ADM transpulmonary gradient could predict postoperative morbidity.

## Methods

In this prospective cohort study, 39 patients undergoing cardiac surgery using CPB were included. Blood was collected before surgery (T0), after induction of anesthesia (T1), after termination of CPB (T2), at ICU arrival (T3) and 3 hours (T4), 6 hours (T5) and 18 hours (T6) after arrival. Pro-ADM was measured with a sandwich immunoassay. Primary endpoints were length of ICU and hospital stay (ICU-LOS, hospital-LOS).

## Results

**Figure 1:**
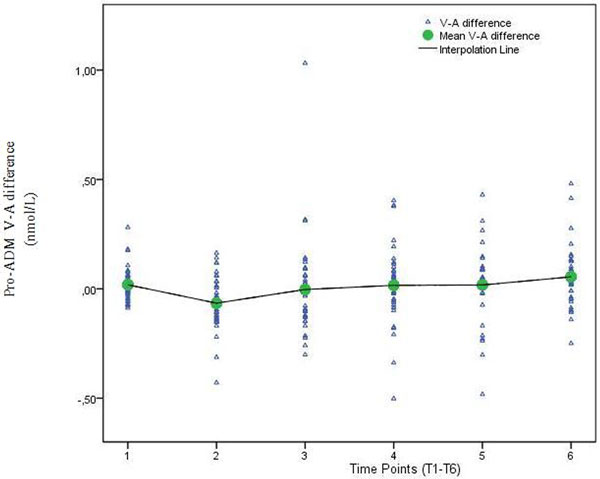
**Pro-ADM transpulmonary gradient at different time points**.

An increase of arterial and venous pro-ADM plasma concentrations was observed after surgery. Immediately after termination of CPB the venous concentration was significantly lower than arterial pro-ADM concentration, but at T6 the venous concentration was significantly higher, indicating a switch from a negative to positive transpulmonary gradient (Figure 1). The pro-ADM venous-arterial difference at T5 was a significant predictor of ICU-LOS (*P *= 0.032) and the difference at T3 was a significant predictor of hospital-LOS (*P *= 0.001).

## Conclusion

We found that the transpulmonary gradient of pro-ADM was a predictor for ICU-LOS and hospital-LOS at T3 and T5, respectively. Pro-ADM might be a promising marker for prediction on outcome of patients undergoing cardiac surgery on CPB. The transpulmonary shift of pro-ADM might be caused by an inflammatory response.
